# Performance of whole blood interferon-γ release assays in SARS-CoV-2 and tuberculosis is age dependent

**DOI:** 10.1007/s15010-025-02613-w

**Published:** 2025-07-30

**Authors:** Tobias Rothoeft, Anna Teresa Hoffmann, Christoph Maier, Robin Denz, Robin Kobbe, Anette Friedrichs, Georg M. N. Behrens, Pia Behrens, Reinhard Berner, Amke Caliebe, Claudia M. Denkinger, Katharina Giesbrecht, Leonhard Hojenski, Olga Hovardovska, Alexandra Dopfer-Jablonka, Olga Iatseniuk, Achim J. Kaasch, Monika Kraus, Lazar Mitrov, Matthias Nauck, Susana Nunes de Miranda, Margarete Scherer, Yvonne Schmiedel, Dana Stahl, Nina Timmesfeld, Nicole Toepfner, Janne Vehreschild, Walter A. Wohlgemuth, Astrid Petersmann, Maria J. G. T. Vehreschild, Folke Brinkmann, The NU(M)KRAINE Study Group

**Affiliations:** 1 University Children’s Hospital, Katholisches Klinikum Bochum, Ruhr-University Bochum, Bochum, Germany; 2 Department of Medical Informatics, Biometry and Epidemiology, Ruhr University Bochum, Bochum, Germany; 3 Institute for Infection Research and Vaccine Development (IIRVD), University Medical Centre Hamburg-Eppendorf, Hamburg, Germany; 4 Department of Infectious Disease Epidemiology, Bernhard Nocht Institute for Tropical Medicine, Hamburg, Germany; 5 Department of Internal Medicine I, Infectious Diseases, University Hospital Schleswig-Holstein, Campus Kiel, Kiel, Germany; 6 Department of Rheumatology and Immunology, Hannover Medical School, Hannover, Germany; 7 German Center for Infection Research, Site Hannover-Braunschweig, Hannover, Germany; 8 Department of Internal Medicine, Infectious Diseases, University Hospital Frankfurt, Goethe University Frankfurt, Frankfurt am Main, Germany; 9 Department of Pediatrics, Faculty of Medicine and University Hospital Carl Gustav Carus, Dresden University of Technology, Dresden, Germany; 10 Institute of Medical Informatics and Statistics, Kiel University and University Hospital Schleswig-Holstein, Campus Kiel, Kiel, Germany; 11 Division of Infectious Disease and Tropical Medicine, Heidelberg University Hospital, Heidelberg, Germany; 12 German Centre for Infection Research, Partner Site Heidelberg, Heidelberg, Germany; 13 University Clinic and Outparticipant Clinic of Radiology, University Hospital Halle, Halle (Saale), Germany; 14 Department of Epidemiology, Helmholtz Centre for Infection Research, Brunswick, Germany; 15 German Centre for Infection Research, TI BBD, Helmholtz Centre for Infection Research, Brunswick, Germany; 16 Faculty of Medicine, Institute of Medical Microbiology and Hospital Hygiene, Otto-Von Guericke University Magdeburg, Magdeburg, Germany; 17 Institute of Epidemiology, Helmholtz Zentrum München, Munich, Germany; 18 Department I of Internal Medicine, Faculty of Medicine and University Hospital Cologne, Center for Integrated Oncology Aachen Bonn Cologne Duesseldorf, University of Cologne, Cologne, Germany; 19 Institute of Clinical Chemistry and Laboratory Medicine, University Medicine of Greifswald, Greifswald, Germany; 20 DZHK (German Centre for Cardiovascular Research), University Medicine, Partner Site Greifswald, Greifswald, Germany; 21 Faculty of Medicine, Institute for Digital Medicine and Clinical Data Science, Goethe University Frankfurt, Frankfurt am Main, Germany; 22 Trusted Third Party of the University Medicine Greifswald, Greifswald, Germany; 23 Institute for Clinical Chemistry and Laboratory Medicine, University Medicine Oldenburg, Oldenburg, Germany; 24 Fraunhofer Institute for Translational Medicine and Pharmacology ITMP, Frankfurt am Main, Germany; 25 Airway Research Center North (ARCN), Member of the German Center for Lung Research (DZL), Lübeck, Germany

**Keywords:** SARS-CoV-2, Immunity, COVID-19, IGRA, T cell, Age dependency, Convalescent, Vaccination

## Abstract

**Introduction:**

A lot of research has been done, mainly on tuberculosis (TB), on the extent to which cellular immune protection as measured by interferon-γ release assays (IGRA) is age-dependent. In a previous study we showed that following an Omicron infection, adolescents with a hybrid immunity had a higher probability of having a reactive SARS-CoV-2-specific IGRA than children. Therefore, we examined in a large group of minors and adults whether age influences cellular immunity as measured by IGRA in TB and SARS-CoV-2.

**Methods:**

Participants were recruited at 13 German study sites between September and December 2022. Cellular immunity was analyzed using SARS-CoV-2 and Tb-specific IGRA and humoral immunity against SARS-CoV-2 by measuring antibodies against spike (S) and nucleocapsid protein. Analysis was done depending on natural (convalescent, not vaccinated) or hybrid immunity (convalescent and vaccinated).

**Results:**

Overall, 1401 adults and 392 minors were included. The amount of interferon-γ released by T cells, as well as the probability of a positive SARS-CoV-2 IGRA (OR 1.022) and a positive Tb IGRA (OR 1.047) were age dependent. Sensitivity of SARS-CoV-2 IGRA in natural immunity was lower in minors (0.45), especially in those less than 5 years (0.29) as compared to adults (0.66).

**Conclusion:**

The interferon-γ response to SARS-CoV-2 infections and/or vaccinations and to Tb infections as measured by IGRA is in quality and quantity dependent on age. The sensitivity of commercially available tests in young children seems to be suboptimal, limiting their use as a diagnostic or research tool in this age group.

**Supplementary Information:**

The online version contains supplementary material available at 10.1007/s15010-025-02613-w.

## Introduction

The adaptive immune response is essential to control and eliminate tuberculosis (Tb) as well as many other infectious agents. A major determinant of the clinical outcome is the cellular immune response mediated by T cells. The most commonly used test to monitor cellular immunity is the interferon-γ release assay (IGRA); an in-vitro diagnostic test used to measure interferon-γ (IFN- γ) released by T cells after stimulation with a specific antigen. The *Mycobacterium tuberculosis* complex (Tb)-specific IGRA was the first commercially available test of this kind used in clinical practice to identify Tb infections [1], now available in the fourth test generation. IGRAs are also used to evaluate specific T-cell immunity after other infections and/or vaccinations, for example SARS-CoV-2 [2, 3]. Furthermore, they are considered to be helpful in monitoring the immune response during immunosuppressive therapy [4, 5].

In a previous study we found that vaccinated adolescents with a hybrid immunity (convalescent and vaccinated) following an Omicron infection had a higher probability of having a reactive SARS-CoV-2-specific IGRA than children with a natural immunity [6]. Due to the design of the study and the relatively small study sample we could not deduce, if this effect was due to a strong bias caused by different vaccination rates or different amounts of antigen used for vaccination in the different age groups. In previous studies using Tb-specific IGRA, younger age was associated with indeterminate IGRA results [7, 8] and lower sensitivity [9], suggesting an influence of the age of the participant on the outcome of these tests in general.

The NU(M)KRAINE study investigated the health status, medical history and vaccination status of Ukrainian war refugees in Germany. While the humoral immune response against SARS-CoV-2 was characterized by presence and level of antibodies against nucleocapsid- and spike protein, cellular immune responses against SARS-CoV-2 and Tb were evaluated by IGRA. The aim of this secondary data analysis was to evaluate age dependency in the cellular immune response as measured by IGRA and to exclude possible influences of co-morbidities and infections.

## Materials and methods

### Study design

As previously described the cross-sectional NU(M)KRAINE study assessed the prevalence of infectious diseases, immunity to vaccine-preventable diseases, and chronic medical conditions in a large cohort of refugees from Ukraine [10, 11]. Two different leading ethics committees evaluated the study protocol, the committee of the Goethe University in Frankfurt for adult participants (reference number 2022-831) and the committee of the Ruhr University in Bochum for the paediatric cohort (registration number 22-7623). Subsequently, the study was approved by all local ethic committees of participating study centres. The study was conducted in accordance with the Declaration of Helsinki.

After informed consent semi-structured interviews were conducted with trained interpreters, a physical examination, and a non-mandatory blood test were performed. All data were recorded in a password protected central databank (SECUtrial, electronic Case Report Form [eCRF]). All laboratory samples were analysed in the University Institute for Clinical Chemistry and Laboratory Medicine Oldenburg and data sets merged after completion of recruitment as described previously [10, 11].

### Antibody measurement

Antibody measurements to determine anti-SARS-CoV-2 antibody titers were conducted using electrochemiluminescence immunoassay (ECLIA, Roche Diagnostics GmbH, Mannheim, Germany). SARS-CoV-2 antibody test against spike (S) and nucleocapsid (NCP) protein were based on IgG and IgM. SARS-CoV-2 spike (S) protein antibodies were assessed quantitatively. Values ≥ 0.8 binding antibody units (BAU)/ml) were considered positive for SARS-CoV-2 spike (S) protein antibodies. Measurements of nucleocapsid (NCP) protein antibodies were assessed qualitatively and considered positive if values were above the assay-specific cut-off index [COI] ≥ 1.0 IU/ml.

### IGRA

The SARS-CoV-2-specific interferon-γ release response was obtained using the QuantiFERON-SARS-CoV-2 assay [2, 6] and the Tb-specific interferon-γ releasing response was obtained using the QuantiFERON®-Tb Gold Plus assay (both DiaSorin GmbH, Dietzenbach, Germany). The results were interpreted according to cut-off values provided by the manufacturer’s specifications.

### Statistics

Raw counts and frequencies were used to describe differences between minors and adults in univariate analysis for categorical variables. Similarly, medians and ranges were used for continuous variables. The main analysis was carried out using logistic regression models for binary endpoints and linear regression models for continuous outcomes. Both types of regression models were calculated with and without adjustment for age and vaccination status. Associated 95% confidence intervals and p-values were also calculated.

Although the main aim of this study was to determine the relationship between age and the reactivity of the IGRA and the amount of interferon-γ as detected by IGRA, the analysis was not pre-registered and is therefore exploratory in nature. Because of this, all analyses have exploratory character; *p*-values are, therefore, only for orientation and have no confirmatory significance. The entire analysis was carried out using the R programming language (version 4.2). Sensitivity of SARS-CoV-2-specific IGRA in not vaccinated participants with proven infection (defined by detection of NCP antibodies and/or S antibodies) was calculated using a standard 2 × 2 table.

## Results

**Fig. 1:**
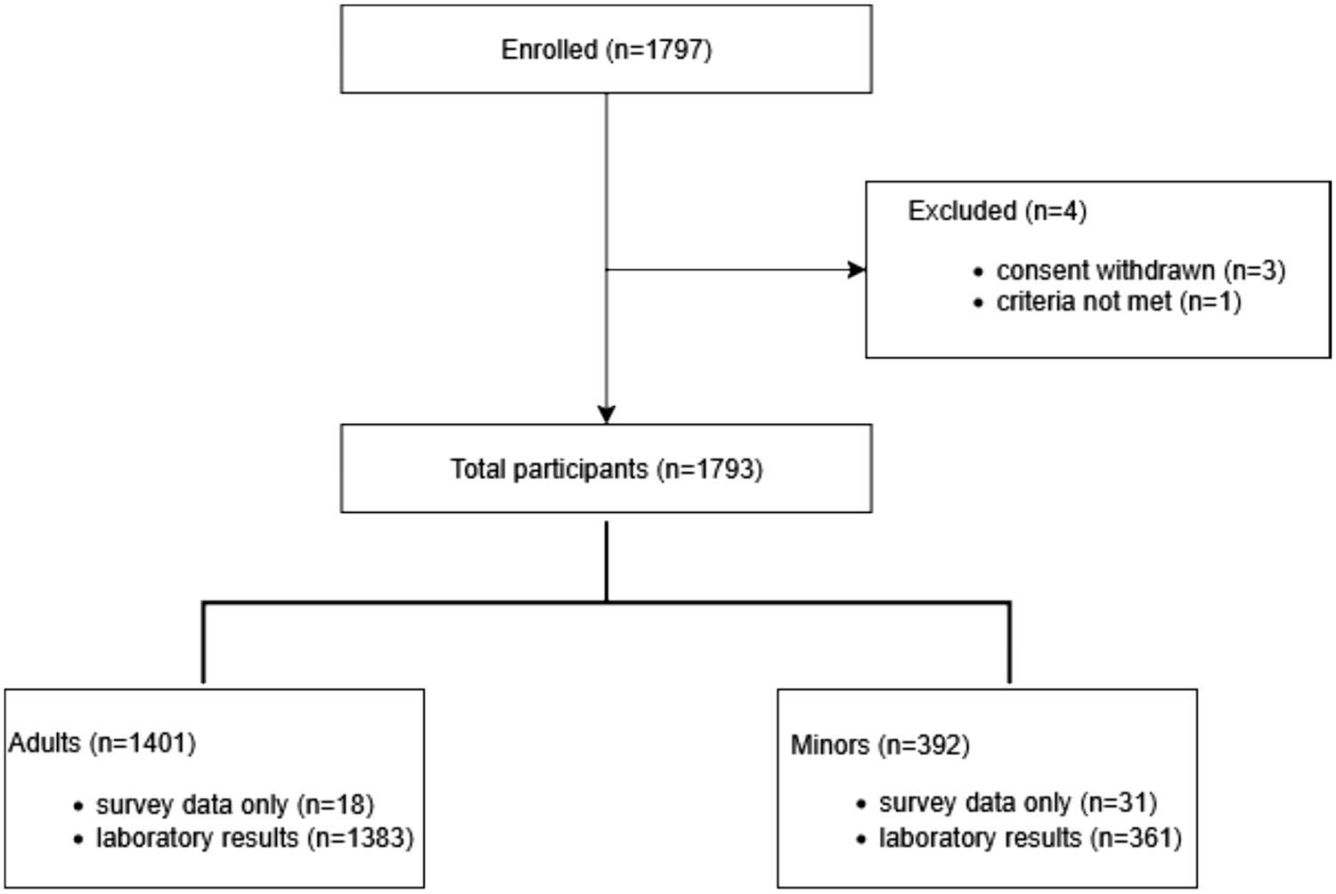
Flowchart of participant recruitment

In total, 1,793 refugees, of which 1401 were adults (> 18 years) and 392 minors (children (0–10 years) and adolescents (11–18 years)) were included in the analysis. Figure 1 shows a flowchart of participant recruitment and procedure. The majority were females (73%, n = 1,307) with a median age of 41 years in adults (range: 18–90). Among 392 included minors (< 18 years), gender was almost equally distributed with 55% females (n = 214) and a median age of 12 years (range: 11 months-18 years).

Overall, 38% (n = 676) of the participants had a history of chronic disease (children 14%, n = 54; adults 44%, n = 622). Adult participants reported most frequently cardiovascular (19%, n = 264), renal (8%, n = 110), rheumatological (7%, n = 101), and pulmonary diseases (4%, n = 50), as well as neurological conditions (6%, n = 84). Chronic infections were reported by 3.7% (n = 67) of adult participants; 14 (1%) reported a chronic hepatitis B, 22 (1.6%) chronic hepatitis C, and 21 (1.5%) reported a HIV infection. Eight participants (0.6%) had a medical history of active Tb, of which four cases were pulmonary Tb [11].

Of the 14% of participating minors with chronic illnesses, most frequently, developmental delays (5.6%, n = 22) and congenital anomalies (4.8%, n = 19) were reported. 1.5% of the minors (n = 6) had either acquired or congenital immunodeficiencies. Active tuberculosis was reported in only two participants (0.5%). 92% of participants had antibodies against Tetanus, 97% had antibodies against HiB and 77% against Measles [10].

The few SARS-CoV-2 vaccinated children had received BNT162b2 in a dose of 10 µg. Adolescents as well as adults were vaccinated with BNT162b2 (30 µg per dose), CoronaVac, JCovden, Spikevax, Sputnik V or Vaxzevria in homologous or heterologous vaccination schemes. Some participants carried no vaccination passports and could not denominate the substance that had been used (Table 1).

**Table 1:** Participants by decade age and self-reported or documented vaccination rate in the corresponding age groups

Age in years	0–10	11–20	21–30	31–40	41–50	51–60	61–70	71–80	81–90
Participants (n)	146	317	212	374	378	179	147	35	5
SARS-CoV-2 vaccinated (n)	3	76	146	236	282	131	96	23	3
SARS-CoV-2 vaccinated (%)	2	24	69	63	75	73	65	66	60

Anti-S-SARS-CoV-2 antibodies were detectable in 99% (n = 1729/1793) and anti-NCP-SARS-CoV-2 antibodies were detectable in 95% (n = 1658/1793). S- and NCP-specific antibody titers as well as the probability of a positive antibody response were not influenced by the age of the participants (Supplemental Figs. 1 and 2). Higher titers of anti-S-antibodies were associated with a higher probability of positive SARS-CoV-2-specific interferon-γ release assays (crude OR: 1.016, 95% CI: 1.012–1.019, *p* < 0.001, per increase of 100 units) (Supplemental Fig. 3). The NCP specific antibody titer height did not correlate with cellular immunity.

**Fig. 2:**
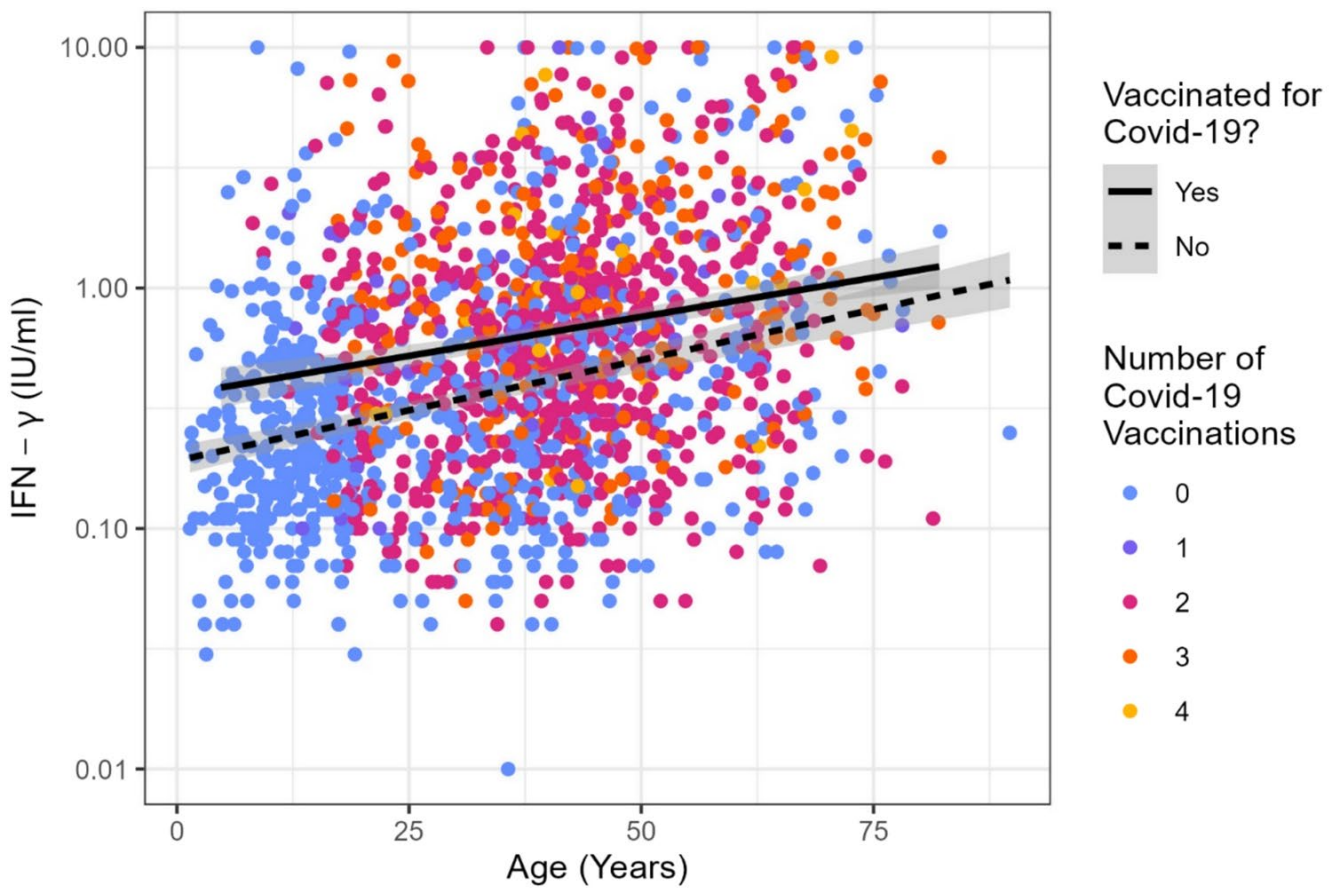
Correlation between age and interferon-γ concentrations detected in SARS-CoV-2-specific IGRA. Shown are results from reactive as well as non-reactive IGRA. Results from indeterminate IGRA were excluded. The black line depicts the mean IFN-γ concentration found in vaccinated participants, the dashed line the results in non-vaccinated participants

**Fig. 3:**
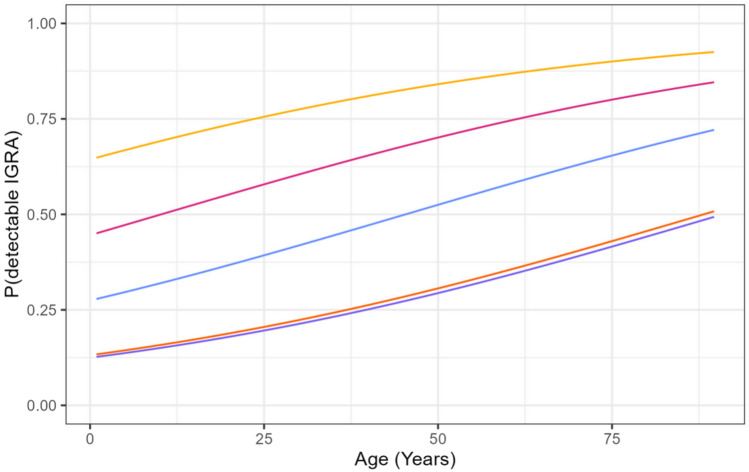
The probability of a positive SARS-CoV-2 IGRA depending on the age of the participants as predicted by a multivariable logistic regression model. The probability of a positive IGRA increased continuously across all age groups, whether the participants had a natural or a hybrid immunity. The yellow line depicts the probability of a positive IGRA in twice vaccinated participants with detectable Spike protein-antibodies, the orange line in twice vaccinated participants without detectable Spike protein- and NCP-antibodies. The magenta, cyan-blue and purple lines show the probability of a positive IGRA in non-vaccinated participants who either had detectable Spike protein- and NCP-antibodies (magenta), only Spike protein-antibodies (cyan-blue) or only NCP-antibodies (purple) (see Supplemental Table 1)

A SARS-Cov-2 IGRA was performed in 1677 participants with positive results in 72.03% (n = 1208) and negative in 27.43% (n = 460). Nine tests (0.54%) had an indeterminate result. All participants with an indeterminate SARS-CoV-2 IGRA either due to a failed mitogen response or due to a high background level of IFN-γ were adults. Five of them had a negative and four also an indeterminate Tb-specific IGRA.

The average IFN-γ response as detected by SARS-CoV-2-specific IGRA increased with age (Fig. 2). This finding was consistent even after adjusting for the number of received vaccinations (adjusted Beta-Coefficient of Age: 0.007, 95% CI: 0.005–0.008, *p* < 0.001, per increase of 1 year). The age effect was present in convalescents and those with a hybrid immunity, albeit the latter had higher IFN-γ levels than convalescents. There also was a strong positive linear association between age and the odds of a positive SARS-CoV-2-specific IGRA (adjusted OR: 1.022, 95% CI: 1.014–1.029, *p* < 0.001, per increase of 1 year). This finding was consistent in all participants (Fig. 3), whether they had a natural (convalescent, not vaccinated) or hybrid immunity (convalescent and vaccinated). The highest probability across all age groups had participants who were vaccinated and also had NCP-antibodies as a sign of hybrid immunity. Those who were only vaccinated and probably had no SARS-CoV-2 infection, as shown by the lack of NCP-antibodies, had the lowest probability of a positive SARS-CoV-2-specific IGRA. Participants with a natural immunity who had detectable Spike protein- and NCP-antibodies also had a higher probability for a positive IGRA than those who had a lacking or partially lacking humoral immune response.

**Fig. 4:**
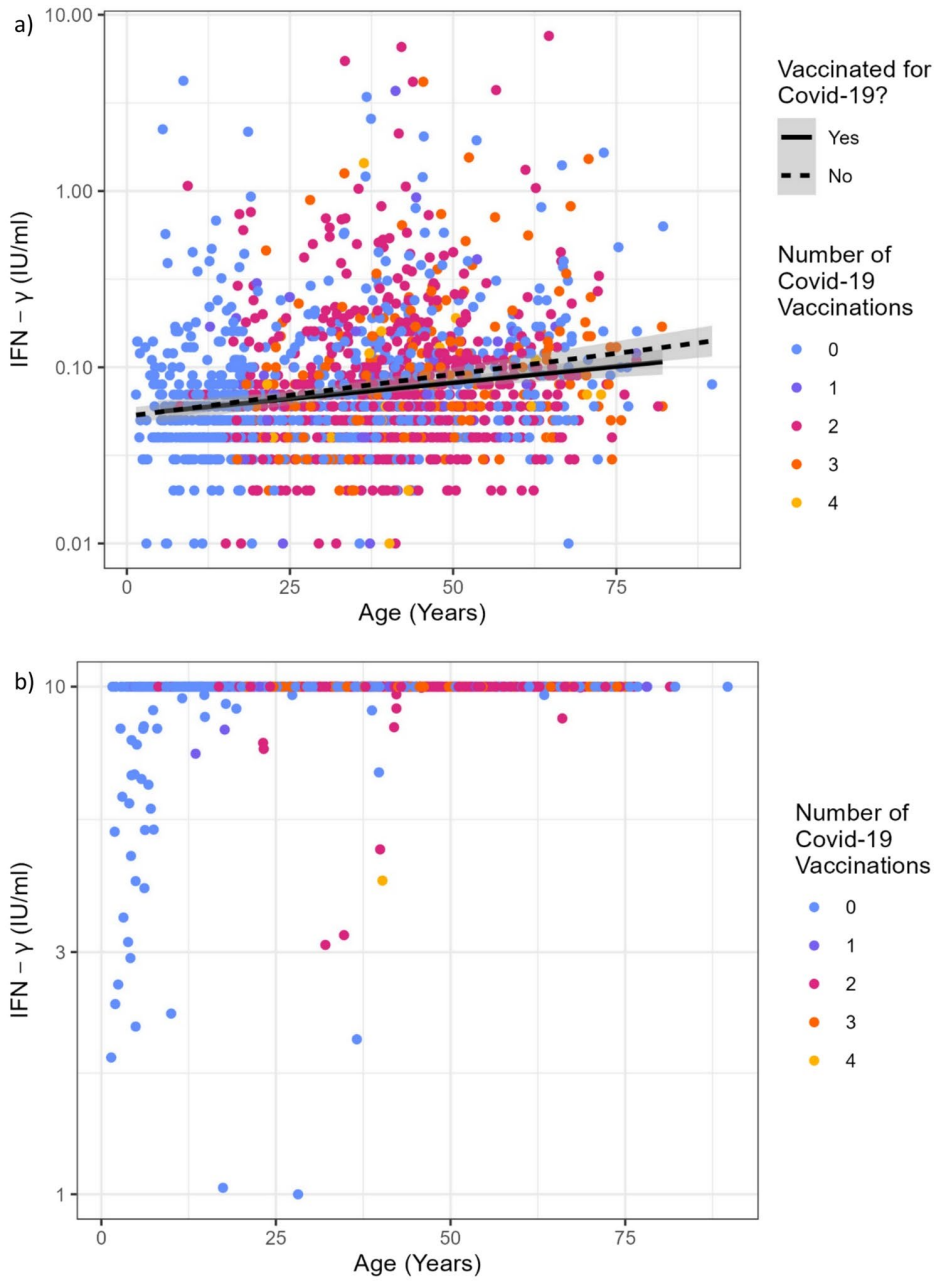
**a** Age dependent amount of IFN-γ in NIL control tubes. The amount of spontaneously released IFN-γ increases with age. The black line depicts the mean IFN-γ concentration found in the probes of vaccinated participants, the dashed line the results in non-vaccinated participants. **b** Age dependent amount of IFN-γ in MITOGEN control tubes. No age-dependent increase can be detected, but most values are above the cut-off of 10 IU/ml. IFN-γ concentrations < 10 IU/ml after mitogen stimulation are mostly detected in children

The amount of IFN-γ detected in the NIL-tubes of SARS-CoV-2-specific IGRA also increased with age. A similar shift could not be detected in the mitogen controls, as most probes contained IFN-γ amounts above the cut-off value of 10 IU/ml. Mitogen controls containing less than 10 IU/ml IFN-γ were mostly found in children (Fig. 4).

A Tb-specific IGRA was performed in 1726 persons. This was positive in 11% (n = 191), negative in 89% (n = 1535) and indeterminate in 0.3% (n = 5). The probability of having a positive Tb-specific IGRA as well as the quantitative amounts of IFN-γ released also increased with age. Higher amounts of IFN-γ detected in SARS-CoV-2-specific IGRA correlated with a higher probability of a positive Tb-specific IGRA (Supplemental Fig. 4). Data were adjusted for age to exclude an age effect by a higher cumulative exposition. The amount of IFN-γ detected in the NIL-tubes of Tb-specific IGRA also increased with age (data not shown).

The sensitivity of SARS-CoV-2-specific IGRA was evaluated in participants with a natural immunity with a proven SARS-CoV-2 infection who had detectable S protein and/or NCP protein specific antibodies. The sensitivity was much lower in children less than 10 years and especially in those younger than 5 years than in adults (Table 2) . In contrast, humoral immunity as measured by antibodies against S and NCP protein were not affected by age (Supplemental Figs. 1 and 2).

**Table 2:** Sensitivity of SARS-CoV-2-specific IGRA in participants with natural immunity. Evaluated were participants who were not vaccinated and had detectable antibodies against S protein and/or NCP protein

	Overall sensitivity	Sensitivity in children < 5 years	Sensitivity in children 5–10 years	Sensitivity in adolescents 11–20 years	Sensitivity in adults > 20 years
	0.59	0.29	0.45	0.56	0.66
95% CI	0.56–0.63	0.13–0.51	0.34–0.57	0.49–0.63	0.61–0.71

## Discussion

This study investigated the age dependency of Tb- and SARS-CoV-2-specific IGRAs in children, adolescents and adults using data from a large cohort of Ukrainian war refugees.

The results obtained by our study are not biased by a random selection of individuals with chronic illnesses, although the proportion of chronically ill or disabled persons was slightly higher than in a German comparison population [11]. More than 92% of participants had antibodies against Tetanus and HiB, proving evidence of a normal humoral immune response. Only 1.5% of participating minors reported an acquired or congenital immunodeficiency, while about 10% of participating adults had a medical condition probably associated with a compromised immune system [6, 12, 13].

The QuantiFERON-SARS-CoV-2 assay was validated [14, 15] in participants infected with SARS-CoV-2 variant B.1.1.7 (Alpha) and its usability was also shown in the Omicron era [16], such that we can also rule out any influence of infections by different subtypes of SARS-CoV-2.

We found an association between positive SARS-CoV-2 IGRA and rising age in the background of both, hybrid and natural immunity. In convalescent participants with only natural immunity, we showed a lower sensitivity of SARS-CoV-2 IGRA in children than in adolescents and adults.

The vaccination of children with lower doses could be a factor influencing our results. Up to an age of 11 years BNT162b2 is dosed with 10 µg mRNA instead of 30 µg (recommendation of the manufacturer). A vaccination with BNT162b2 in lower doses (2 × 10 µg) is associated with a decrease in IFN-γ production after heterologous bacterial, fungal and viral re-stimulation 28 days after vaccination compared to pre-vaccination and remained decreased after viral re-stimulation after six months [17]. In line with this observation we found a lower probability of a positive IGRA in children compared to adolescents or adults. But as the probability of a positive SARS-CoV-2-specific IGRA further increases with age even after adolescence and in both, vaccinated as well as in non-vaccinated individuals, the different vaccination schemes seem not to offer a sufficient explanation for the phenomena described here. In the adult cohort described here several different COVID-19 vaccines were used either in homologous or heterologous fashion. For some of the schemes used in this daily-life cohort no immunological data exist. In general, most homologous as well as heterogeneous vaccination approaches elicit a robust cellular and humoral immune response [12, 13, 18].

The clinical course of COVID-19 in children is significantly less severe than in adults [19–22], a fact that may influence the less pronounced cellular immune response in children. In a previous study, we found that younger children with asymptomatic infections were less likely to have a reactive IGRA than adolescents after a symptomatic SARS-CoV-2 infection [6]. In general, a reactive IGRA is described to be less probable in persons with a natural immunity as in vaccinated or those with a hybrid immunity [16]. As the probability of a positive IGRA increased continuously across all age groups whether they had a natural or hybrid immunity, the different severity of the clinical course does not sufficiently explain our results.

**Fig. 5:**
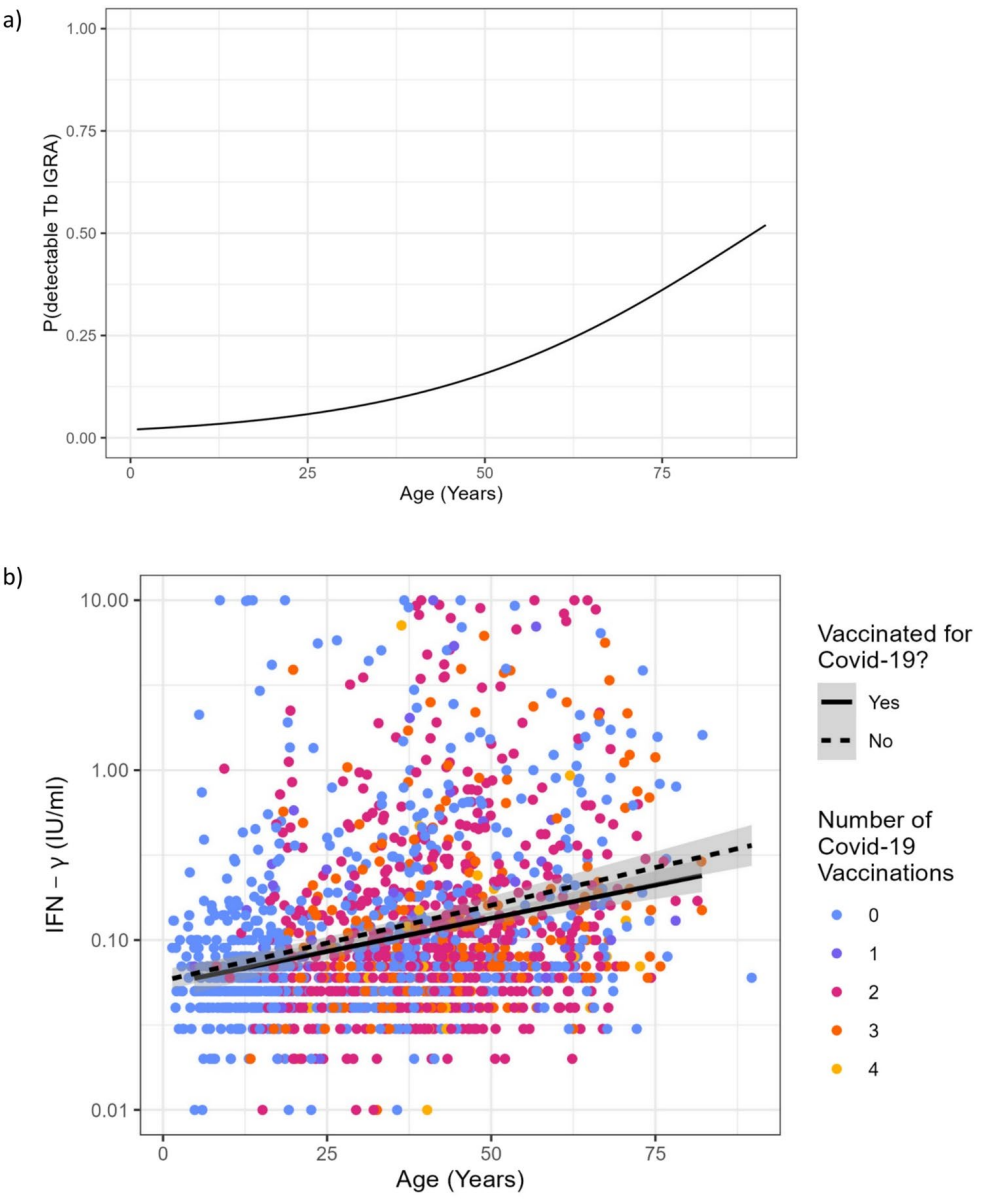
**a** The probability of a positive Tb-specific IGRA depending on the age of the participants, as predicted by a univariable logistic regression model. The probability of a positive IGRA increased continuously across all age groups. (see Supplemental Table 2). **b** Correlation between age and interferon-γ concentrations detected in Tb-specific IGRA. Shown are results from reactive as well as non-reactive IGRA. Results from indeterminate IGRA were excluded. The black line depicts the mean IFN-γ concentration found in the samples of SARS-CoV-2 vaccinated participants, the dashed line the results in non-vaccinated participants

IGRA are regarded to be a highly specific tool to detect SARS-CoV-2-specific T cell response to infection as well as vaccination, but their sensitivity decreases over time [14]. We could not specify the time of infection in most participants, but should rule out a systemic bias here; it is highly unlikely that all participating children and adolescents would have had a longer interval between infection and/or vaccination and testing than the adults. Since this comparative analysis was only done with those who were vaccinated twice (Fig. 5), we can exclude booster effects as influencing factors.

The phenomenon of a higher rate of indeterminate Tb-specific IGRA in children than in adults [7, 8, 23, 24] has already been described. The etiology of the failed positive mitogen control responsible for the indeterminate IGRA results is likely to be multi-factorial, but may be associated with poor health [24, 25], as lymphocyte activation by PHA is a surrogate marker for the immune status of the individual. We could not reproduce this finding of a higher rate of indeterminate IGRA in children, neither in SARS-CoV-2-specific IGRA nor in Tb-specific IGRA, but then again, we were examining a cohort with no co-morbidities such as malaria, malnourishment, or other chronic illnesses. We did find a lower amount of interferon-γ release in the mitogen control in children, which probably correlates with the previous studies suggesting a diminished interferon-γ secretion response to PHA [8, 26]. The amount of interferon-γ was not reduced to the point of a failed positive control.

In our cohort, the probability of a positive Tb-specific IGRA also increased with age, but one of the main factors positively associated with Tb infection in high-burden settings includes increasing age [27, 28]. Therefore, we cannot prove by our data if this is an age dependent effect of the immune system or the cumulative exposure to Tb as we are lacking comprehensive microbial results as well as systematic follow-up data due to the design of this study. However, previous results by the multicentre PTBNET study recently reported a suboptimal sensitivity of Tb-specific IGRA in children and adolescents [9] that were also suggesting an at least partly age dependent effect.

In participants with a high amount of IFN-γ detected in SARS-CoV-2-specific IGRA, the probability of a positive Tb-specific IGRA was also increased. These higher amounts of IFN-γ are especially detected in those with a hybrid immunity. If the combined and repetitive immune stimulation by infection and vaccination also unspecifically activates T cells specific for other antigens remains speculative; at least in animal models, viral infections increase the IFN-γ secretion of BCG-specific T cells [29]. Significantly higher IFN-γ concentrations in the mitogen controls of Tb-specific IGRA after COVID-19 vaccination or infection have already been described in the literature [30, 31], making such an unspecific activation effect a possibility.

Since most of the children were unvaccinated, we calculated the sensitivity of the SARS-CoV-2-specific IGRA in the group of unvaccinated convalescent participants in order to be able to include the largest cohort in this calculation. The sensitivity was much lower in children less than 10 years and especially in those younger than 5 years. The reasons for the age dependency of the IGRA are probably caused by age-related changes in the immune system.

Although SARS-CoV-2 infections in children generate Spike protein-specific CD4^+^ and CD8^+^ T-cell responses [32], a lower frequency of IFN-γ^+^ CD4^+^ T cells in pediatric than in adult SARS-CoV-2 participants [33] has been reported. An age dependency with a lower frequency of SARS-CoV-2-specific T cells in children than in adults in study groups infected with the ancestral strain utilizing flow cytometry to detect antigen-specific T cells [34–36] has also been described in the literature.

Contrary to these findings in younger children previous studies have shown that children older than 5 years and adults with mild SARS-CoV-2 infection had comparable numbers of virus-reactive CD4^+^ T cell responses [37]. Another possible explanation for the lower sensitivity in children despite the comparable numbers of virus-reactive CD4^+^ T cells may be the bystander activation of IFN-γ producing CD4^+^ [38, 39] and CD8^+^ memory T cells [40] by cytokines or via TLR-stimulation which is a well described phenomenon. This phenomenon is much less pronounced in naïve T cells [38]. As the number of naïve T cells decreases whereas the number of memory T cells increases with age [41, 42], showing a shift towards memory based immunity, this naivety effect may explain our findings in children. This effect could also explain the lower amount of interferon- γ release in the mitogen control in children, as children specifically have high numbers of naïve T cells in peripheral blood [42]; although naïve T cells may produce IFN-γ upon stimulation, this process usually takes several days [43, 44] instead of the 24 h used in the mitogen controls in IGRA.

We also found a higher background IFN-γ secretion in unstimulated cells, that was also age dependent. In previous studies using Tb-specific IGRA performed before and after COVID-19 vaccination or comparing Tb-specific IGRA before and after the pandemic, no changes in spontaneous IFN-γ secretion could be detected [30, 31]; on the contrary, spontaneous IFN-γ secretion tended to be lower following vaccination [31]. Therefore, we would assume the changes observed in the background secretion of IFN-γ secretion in our cohort as purely age dependent and not as an unspecific activation by COVID-19 vaccinations and or infections. Importantly, this higher background activity was found in the NIL-control of SARS-CoV-2-specific IGRA as well as Tb-specific IGRA which is plausible as the method is the same.

We were able to replicate the results of our earlier pilot study and show that humoral immunity against SARS-CoV-2 is not age dependent and that extremely high S-antibody titers correlate with cellular immunity [6]. Titers of this magnitude were only found in adolescents and adults with a hybrid immunity. If this effect is only caused by vaccination or is also dependent on the age of the participants cannot be deduced from this study.

In summary, the increasing probability for a positive IGRA with increasing age in hybrid and natural immunity and the lower sensitivity in natural immunity in children may be the result of cumulative effects of a lower number of specific T cells and decreased bystander activation due to lower numbers of memory T cells. These effects point towards a relatively lower applicability of IGRA in especially in children younger than one year. [45]

## Conclusion

We have identified differences in the cellular immune responses to SARS-CoV-2 infection as detected by IFN-γ release assays between pediatric, adolescent and adult participants with COVID-19. Younger age was associated with lower amounts of IFN-γ detected by IGRA as well as a higher probability of a negative test and a lower sensitivity, but not with a higher probability of an indetermined result. The spontaneous IFN-γ release as detected by the NIL-controls also increased with age. These differences should be taken into account when using these IGRA for disease specific diagnostic or research purposes and might be appliable to all IGRA based diagnostic procedures.

### Limitations

Limitations include the fact that the majority of the cohort was from Ukraine and there was insufficient representation of ethnic and geographical variability to draw firm conclusions about the generalizability of these data. It should be expected that the increase in positive IGRAs would reverse at some point with increasing age due to senescence of the immune system. As few participants older than 70 years were included in the NU(M)KRAINE study we might have missed this point. The sensitivity of SARS-CoV-2-specific IGRA could only be calculated in convalescent participants, as most children were not vaccinated.

As a Tb-specific IGRA does not allow a differentiation between LTBI, disease or previously experienced disease and the fact that we have no comprehensive follow-up of study participants we cannot assign all our findings in the Tb-specific IGRA as the results of age dependent changes in the immune system in contrast to our findings in the SARS-CoV-2-specific IGRA.

Meanwhile the strengths of this study, which include the size of the cohort, the inclusion of participants of all ages, the standardization and extension of the investigations in our eyes outweighs the mentioned limitations.

## Supplementary Information

Below is the link to the electronic supplementary material.

Supplementary file1 (PDF 241 KB)

Supplementary file2 (PDF 181 KB)

Supplementary file3 (PDF 100 KB)

Supplementary file4 (PDF 118 KB)

Supplementary file5 (DOCX 15 KB)

## Data Availability

No datasets were generated or analysed during the current study.
