# Supplementary material for: Performance of whole blood interferon-γ release assays in SARS-CoV-2 and tuberculosis is age dependent
Source: Infection. 2025 Jul 30;53(6):2669–80. doi: 10.1007/s15010-025-02613-w (PMC12675746; doi:10.1007/s15010-025-02613-w)
Supplement: Supplementary file 2 — Supplementary file2 (PDF 181 KB) [file 15010_2025_2613_MOESM2_ESM.pdf]

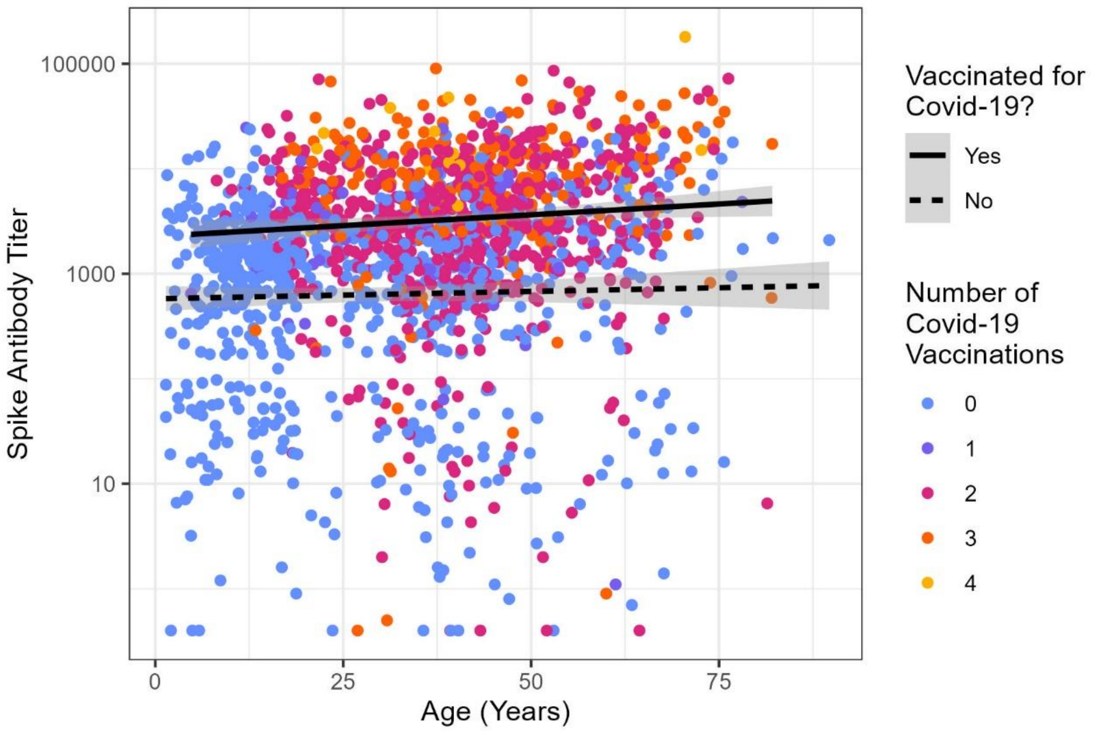

Supplemental Figure 2: Spike protein-antibody titers were independent of the age of the participants.
