# Supplementary material for: Performance of whole blood interferon-γ release assays in SARS-CoV-2 and tuberculosis is age dependent
Source: Infection. 2025 Jul 30;53(6):2669–80. doi: 10.1007/s15010-025-02613-w (PMC12675746; doi:10.1007/s15010-025-02613-w)
Supplement: Supplementary file 4 — Supplementary file4 (PDF 118 KB) [file 15010_2025_2613_MOESM4_ESM.pdf]

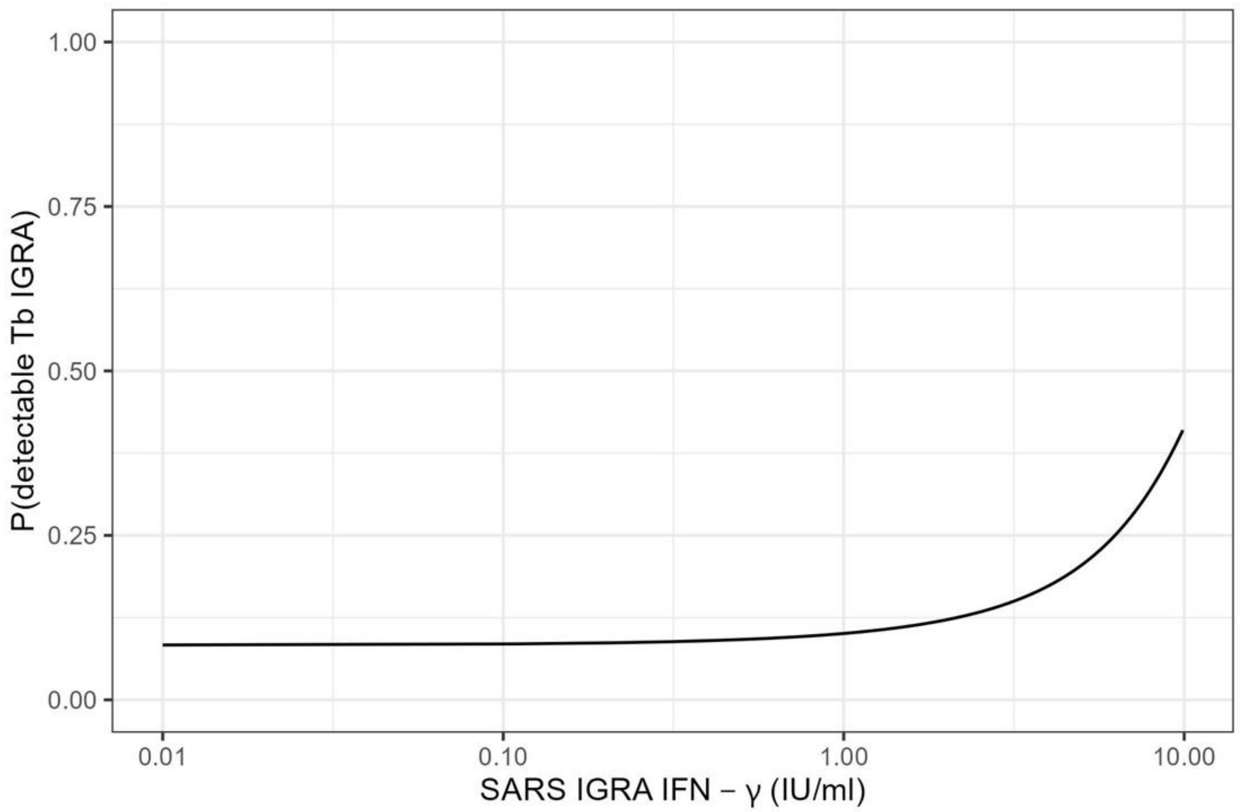

Supplemental Figure 4: High amounts of IFN-γ detected in SARS-CoV-2-specific IGRA correlated with an increased probability of a positive Tb-specific IGRA. Data were adjusted for age.
