# Supplementary material for: Performance of whole blood interferon-γ release assays in SARS-CoV-2 and tuberculosis is age dependent
Source: Infection. 2025 Jul 30;53(6):2669–80. doi: 10.1007/s15010-025-02613-w (PMC12675746; doi:10.1007/s15010-025-02613-w)
Supplement: Supplementary file 5 — Supplementary file5 (DOCX 15 KB) [file 15010_2025_2613_MOESM5_ESM.docx]

| **Characteristic** | **exp(Beta)** | **95% CI**^1^ | **p-value** |
| --- | --- | --- | --- |
| age | 1.022 | 1.014, 1.029 | 0.000 |
| factor(covid_Vacc_num) |  |  |  |
| 0 | — | — |  |
| 1 | 2.638 | 1.336, 5.208 | 0.005 |
| 2 | 2.250 | 1.730, 2.928 | 0.000 |
| 3 | 3.821 | 2.491, 5.862 | 0.000 |
| 4 | 2.975 | 0.854, 10.36 | 0.087 |
| ncp_ab_reac |  |  |  |
| nreac | — | — |  |
| reac | 2.123 | 1.249, 3.606 | 0.005 |
| spike_ab_reac |  |  |  |
| nreac | — | — |  |
| reac | 5.637 | 1.355, 23.45 | 0.017 |
| ^1^CI = Confidence Interval | | | |

*Supplemental Table 1: Statistical model for the calculation of the probability of a positive SARS-CoV-2-specific IGRA depending on the age of the participants, as predicted by a univariable logistic regression model.*

| **Characteristic** | **exp(Beta)** | **95% CI**^1^ | **p-value** |
| --- | --- | --- | --- |
| age | 1.047 | 1.037, 1.057 | 0.000 |
| covid_Vacc_num | 0.870 | 0.760, 0.996 | 0.044 |
| ^1^CI = Confidence Interval | | | |

*Supplemental Table 2: Statistical model for the calculation of the probability of a positive Tb-specific IGRA depending on the age of the participants, as predicted by a univariable logistic regression model.*
